# Ekbom Syndrome: A case report and literature review

**DOI:** 10.1192/j.eurpsy.2025.2234

**Published:** 2025-08-26

**Authors:** P. Martínez Gimeno, L. Rodríguez Andres, C. Alario Ruiz, O. Martín Santiago, B. Arribas Simón, M. P. Pando Fernández, M. Andreo Vidal, M. Calvo Valcarcel, B. Rodríguez Rodríguez, M. Fernández Lozano, M. D. L. Á. Guillén Soto, A. Aparicio Parra, M. Ríos Vaquero, G. Lorenzo Chapate, A. Monllor Lazarraga, L. R. Vazquez, F. J. Gonzalez Zapatero, L. Del Canto Martínez

**Affiliations:** 1 Hospital Clínico Universitario Valladolid, Valladolid, Spain

## Abstract

**Introduction:**

Ekbom’s syndrome is a clinical term for delusional parasitosis, a condition characterized by the belief that one’s skin is infested by invisible parasites. Delusional infestation is a rare psychiatric disorder, is more common in the elderly, particularly in postmenopausal females. Psychiatric interventions are usually rejected by these patients and long-term treatments are frequently abandoned, they usually seek care from dermatologists. It is advocated to form a liaison between dermatology and psychiatry to ensure a full range of differential diagnoses, in order to form the most suitable management plan.

**Objectives:**

The objetive of this case is to illustrate the severity of Ekbom’s syndrome, providing detailed clinical information and highlighting the challenges in treatment.

**Methods:**

The following patient will be presented, doing a thorough systematic bibliography review.

**Results:**

A 54-year-old female patient describes a clinical history of three years of visual hallucinations and generalized pruritus since a family weekend at a countryside house. She reported that, for the past three years, she has experienced itching all over her body and has occasionally seen “bugs” on her body that she believes to be fleas. She mentioned having been diagnosed with “scabies” and “seborrheic dermatitis”.Despite these diagnoses, her father noted that for the past year, the patient has been extremely anxious, spending hours examining her hair and skin, washing repeatedly, and searching for “bugs.” In recent weeks, she refused to eat.Throughout her stay in the unit attended therapy regularly, and participated actively. A psychopharmacological adjustment was made, starting with Abilify at 15 mg/day, which was well-tolerated and effective. A dermatology consult ruled out dermatological pathology. Over the days, a reduction in anxiety and partial improvement in somatic complaints were observed. As the patient’s condition improved, she committed to continuing with the treatment and attending mental health team consultations with her referring psychiatrist.

**Conclusions:**

Delusional infestation is a serious and uncommon disorder that endangers the patients and the people around them, and can be complicated with secondary somatic complications, often requiring involvement of different medical specialists.The treatment is long and complicated, the effectiveness of pimozide, aripiprazol or risperidone for the Ekbom syndrome has been documented in the literature. In our case, we decided to introduce aripiprazol. The management of these patients requires a multidisciplinary approach between dermatologists and psychiatrists, as they often refuse treatment. Consultation and collaboration between both specialties are essential to ensure timely referral. Additionally, it is crucial for general physicians to have greater awareness of these conditions, perform early recognition, maintain good rapport with patients, and provide empathetic treatment.

**Disclosure of Interest:**

None Declared

